# Impact of a Point-of-Care Ultrasound Training Program on the Management of Patients With Acute Respiratory or Circulatory Failure by In-Training Emergency Department Residents (IMPULSE): Before-and-After Implementation Study

**DOI:** 10.2196/53276

**Published:** 2025-03-03

**Authors:** Sandra Bieler, Stephan von Düring, Damien Tagan, Olivier Grosgurin, Thierry Fumeaux

**Affiliations:** 1 Médecin cheffe, Service des Urgences, Hôpital de Sion, Sion, 1950, Switzerland; 2 Faculté de Médecine de l’Université de Genève, Hôpitaux Universitaires de Genève, Genève, Switzerland; 3 Service des Soins critiques, Hôpital Riviera Chablais, Rennaz, Switzerland; 4 Service de médecine interne générale et Service des Urgences, Hôpitaux Universitaires de Genève, Genève, Switzerland; 5 Hirslanden Geneva Clinics, Geneva, Switzerland

**Keywords:** point-of-care ultrasonography, training program, emergency department, acute respiratory failure, acute circulatory failure

## Abstract

**Background:**

Due to its diagnostic accuracy, point-of-care ultrasound (POCUS) is becoming more frequently used in the emergency department (ED), but the feasibility of its use by in-training residents and the potential clinical impact have not been assessed.

**Objective:**

This study aimed to assess the feasibility of implementing a structured POCUS training program for in-training ED residents, as well as the clinical impact of their use of POCUS in the management of patients in the ED.

**Methods:**

IMPULSE (Impact of a Point-of Care Ultrasound Examination) is a before-and-after implementation study evaluating the impact of a structured POCUS training program for ED residents on the management of patients admitted with acute respiratory failure (ARF) and/or circulatory failure (ACF) in a Swiss regional hospital. The training curriculum was organized into 3 steps and consisted of a web-based training course; an 8-hour, practical, hands-on session; and 10 supervised POCUS examinations. ED residents who successfully completed the curriculum participated in the postimplementation phase of the study. Outcomes were time to ED diagnosis, rate and time to correct diagnosis in the ED, time to prescribe appropriate treatment, and in-hospital mortality. Standard statistical analyses were performed using chi-square and Mann-Whitney *U* tests as appropriate, supplemented by Bayesian analysis, with a Bayes factor (BF)>3 considered significant.

**Results:**

A total of 69 and 54 patients were included before and after implementation of the training program, respectively. The median time to ED diagnosis was 25 (IQR 15‐60) minutes after implementation versus 30 (IQR 10‐66) minutes before implementation, a difference that was significant in the Bayesian analysis (BF=9.6). The rate of correct diagnosis was higher after implementation (51/54, 94% vs 36/69, 52%; *P*<.001), with a significantly shorter time to correct diagnosis after implementation (25, IQR 15‐60 min vs 43, IQR 11‐70 min; BF=5.0). The median time to prescribe the appropriate therapy was shorter after implementation (47, IQR 25‐101 min vs 70, IQR 20‐120 min; BF=2.0). Finally, there was a significant difference in hospital mortality (9/69, 13% vs 3/54, 6%; BF=15.7).

**Conclusions:**

The IMPULSE study shows that the implementation of a short, structured POCUS training program for ED residents is not only feasible but also has a significant impact on their initial evaluation of patients with ARF and/or ACF, improving diagnostic accuracy, time to correct diagnosis, and rate of prescribing the appropriate therapy and possibly decreasing hospital mortality. These results should be replicated in other settings to provide further evidence that implementation of a short, structured POCUS training curriculum could significantly impact ED management of patients with ARF and/or ACF.

## Introduction

Acute respiratory failure (ARF) and acute circulatory failure (ACF) are common causes of emergency department (ED) admissions and are associated with significant morbidity, mortality, and ED resource use. Timely and appropriate management can reduce these outcomes but depends on an efficient diagnostic workup [1]. In a high proportion of EDs around the world, patients received first-line treatment by junior in-training physicians. Traditionally, the workup is guided by history taking and physical examination, which have been shown to be inaccurate in the ED, particularly when performed by less experienced physicians [2-4]. Basic laboratory and imaging tests are often supplemented with more advanced modalities, such as transthoracic echocardiography or computed tomography (CT), at the expense of increased ED length of stay, resource use, and potential adverse events [5-7]. Point-of-care ultrasound (POCUS), performed by nonradiologists or noncardiologists, is a noninvasive bedside diagnostic tool that has been shown to be highly accurate in identifying the etiologic cause of ARF or ACF, with no significant side effects [8-20]. POCUS is now included in many training programs for emergency physicians [21-27]. However, it is still unclear if the diagnostic accuracy of POCUS translates into a clinically relevant difference in patient outcomes [18,28-33]. Despite these limitations, the American College of Physicians guidelines recommend the use of POCUS in addition to standard diagnostic procedures in patients with acute dyspnea [34,35]. In most of the published studies, POCUS was performed by trained experts who were not directly responsible for the patient and were often blinded to clinical data, which does not reflect real-life conditions where patients are initially managed by junior or in-training residents.

We designed the IMPULSE (Impact of a Point-of-Care Ultrasound Examination) study to evaluate the feasibility and impact of implementing a structured POCUS training program for in-training ED residents in the first-line management of patients admitted for ACF and/or ARF. A before-and-after implementation study design was chosen to avoid the methodological problems associated with blinding and randomization in a single-center study [35].

## Methods

### Study Design and Intervention

IMPULSE is a single-center, before-and-after, observational, implementation study of a structured POCUS training program for ED residents (first or second year of internal medicine training) at a regional hospital (Hôpital de Nyon, Switzerland). During the preimplementation period (phase 1), patient management was unchanged, and POCUS could only be performed on demand by trained attending physicians as part of the standard ED management implemented since 2010. Only 1 in-training ED resident per 12-hour shift participated in the study.

During the intervention phase, a group of residents in training (first and second year after graduation) were enrolled in the AURUS (Association des urgentistes et réanimateurs intéressés à l’ultrasonographie) training program, organized into 3 steps and in accordance with the European Society of Intensive Care Medicine consensus document [36-38]:

- A 20-hour, web-based course on general principles of ultrasound as well as theoretical and practical aspects of image acquisition and interpretation in transthoracic, cardiac, vascular, pulmonary, and abdominal POCUS [39]: The module includes a formal assessment of knowledge through a multiple-choice questionnaire, which must be completed to proceed to the next step.
- An 8-hour, practical, hands-on session in which POCUS examinations are performed on healthy volunteers and simulators in groups of 3 students under the supervision of an instructor, focusing on the technical aspects of obtaining interpretable images: The session includes a formal assessment of image acquisition and interpretation skills. This assessment is mandatory to proceed to the next step.
- The practice of at least 10 directly supervised POCUS full examinations, performed under real conditions in the ED: This includes a formal assessment of the ability to acquire, interpret, and integrate good-quality images into clinical management.

**Figure 1.**
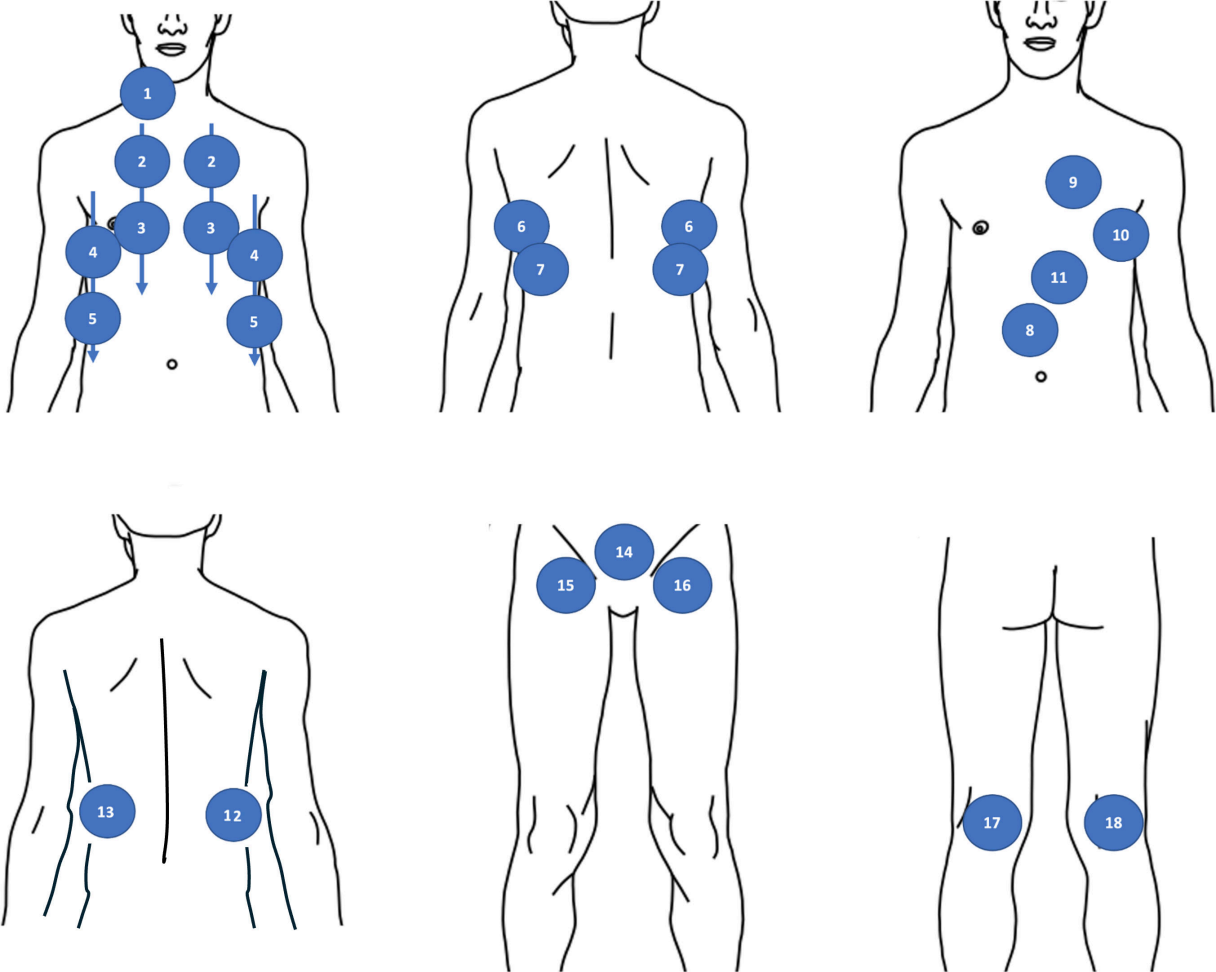
Point-of-care ultrasound (POCUS) protocol evaluating specific sonographic signs: (1) internal jugular vein; (2) to (5) anterior pulmonary view or anterior axillary line view; (6) and (7) posterobasal pulmonary view; (8) inferior vena cava; (9) parasternal short- and long-axis cardiac views; (10) apical four-chamber cardiac view; (11) subcostal cardiac view; (12) hepatorenal space; (13) splenorenal space; (14) suprapubic view; and (15) to (18) femoropopliteal veins.

**Figure 2.**
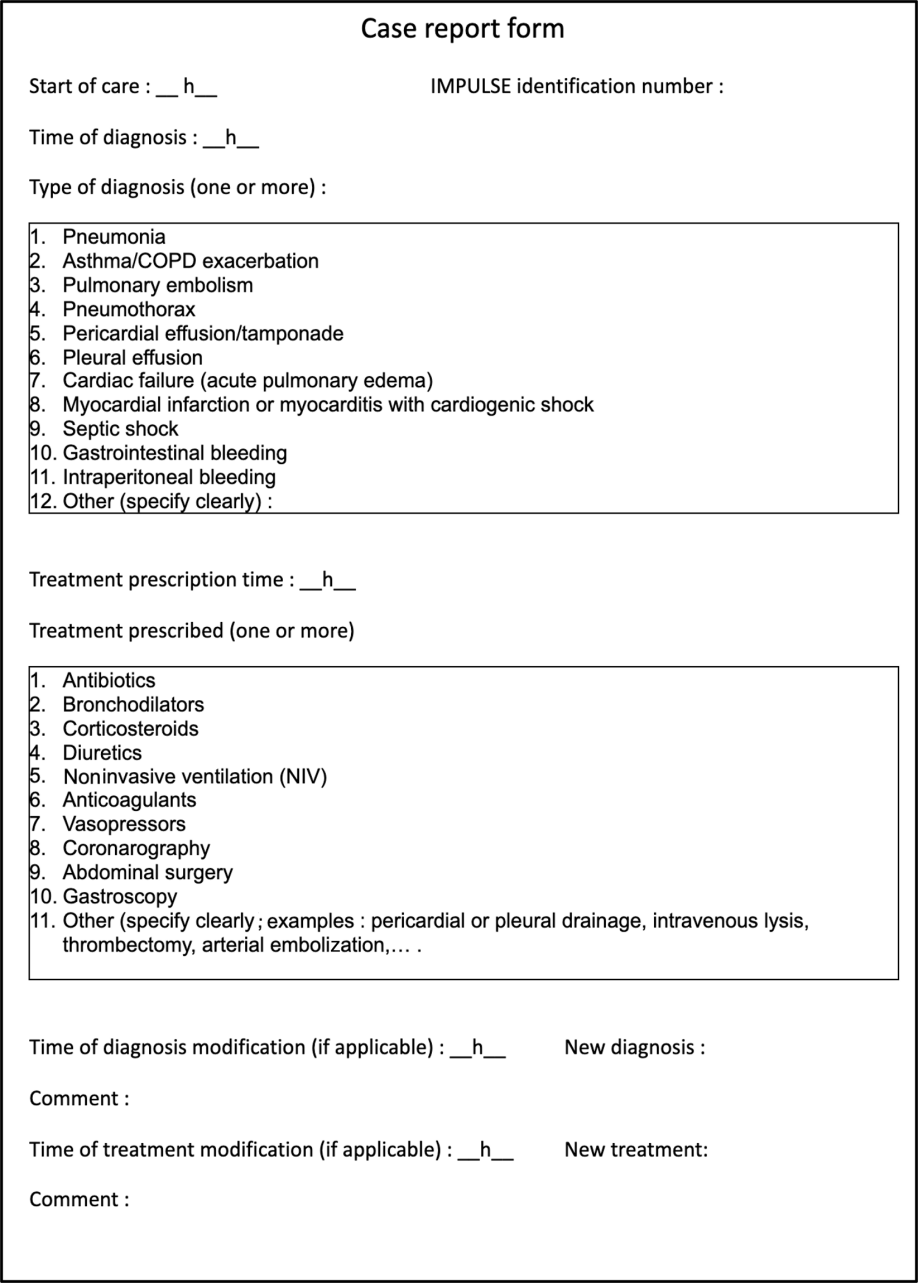
Case report form (adapted from the original form in French). COPD: chronic obstructive pulmonary disease; IMPULSE: Impact of a Point-of-Care Ultrasound Examination.

At the end of the training process, residents who met all training objectives were enrolled in the postimplementation phase (phase 2). Similar to phase 1, only 1 ED resident per shift participated in the study. A Sparq Ultrasound System (Philips AG Healthcare) was used for all POCUS examinations, which were performed with a 4‐12 MHz linear probe and a 1‐4 MHz phased array probe. POCUS was requested to be performed as soon as possible on all enrolled patients, in parallel with the clinical evaluation and according to a standardized protocol evaluating 18 specific sonographic signs (Figure 1), looking for echographic signs of pulmonary embolism, left heart failure, hypovolemic state, tamponade, pneumonia, pneumothorax, or abdominal disease. All POCUS images were recorded, and a standardized case report form was completed by the resident (Figure 2). All images were mandatorily reviewed by a POCUS-trained attending physician, directly or subsequently, to confirm the findings.

All other diagnostic procedures were used at the discretion of the clinician, including a basic POCUS performed by the attending physician and an advanced ultrasound performed by a fully trained radiologist or cardiologist.

### Patient Inclusion and Exclusion Criteria

In both phases, all consecutive adult patients (aged ≥18 years) presenting with ARF and/or ACF were screened for inclusion in the study. ARF was defined by (1) the presence of either signs of respiratory distress or a respiratory rate greater than 20 breaths/min and (2) an oxygen saturation measured using pulse oximetry of <92% on room air or the need to administer oxygen to maintain a saturation of ≥92%. ACF was defined by (1) the presence of a systolic blood pressure <90 mm Hg and (2) clinical signs of hypoperfusion (agitation or altered consciousness, skin mottling, or oliguria) or hyperlactatemia (>2.0 mmol/L).

Exclusion criteria were a known or immediate diagnosis (such as ST-elevation myocardial infarction or referral for an externally determined diagnosis), the need for immediate lifesaving measures (such as cardiopulmonary resuscitation), trauma, palliative care, and patient refusal of care.

In order to preserve the organization of the ED and to favor the admission of patients for whom uninterrupted care seemed likely, the final admission of patients and the start of observation were left to the discretion of the attending physician, based on his or her assessment of the ED situation and workload.

### Data Collection

On a standardized case report form, the ED resident recorded various times (start of observation, time of diagnosis, start of diagnosis-specific therapy, and end of ED stay). Diagnoses and therapies were also reported according to a specified list (Figure 1). The participating resident was equipped with an audio recorder, which was started at first contact with the patient. All recordings were kept confidential only to the investigators, who analyzed them to verify the written data reported. Based on these data, the time to diagnosis; time to prescription of targeted, appropriate treatment; and length of stay in the ED were calculated and rounded to 5-minute intervals. The hospital discharge summary was retrospectively analyzed to compare the diagnosis made during the ED stay with the final hospital diagnosis and to assess in-hospital mortality.

### Statistical Analysis

All data were analyzed with the free, open-source JASP tool (University of Amsterdam). Median and IQR values are reported for descriptive statistics of continuous variables, and absolute numbers and proportions are reported for categorical variables. Differences in proportions of categorical variables between phases were analyzed by chi-square test, with a significant level set at *P*<.05. Differences in continuous variables and time intervals between phases were analyzed with a Mann-Whitney *U* test, completed by a Bayesian approach. For this analysis, the alternative hypothesis was that the time intervals would be greater in phase 1 than in phase 2, with a prior probability described by a Cauchy distribution centered around zero and with a width parameter of 1.00. This width parameter was chosen after an equivalence, Bayesian, independent-samples (2-tailed) *t* test analysis and corresponds to a probability of 50% that the effect size lies between −1.000 and 1.000. The statistical significance of the Bayesian analysis was expressed with the Bayes factor (BF), where a value between 3 and 10 is considered moderate evidence, and a value over 10 represents strong evidence. For hospital mortality comparison between the 2 phases, a Bayesian analysis was also performed, with an independent binomial analysis, with fixed rows.

### Ethical Considerations

The study was approved by the regional ethics committee (Commission Cantonale d’Ethique du Canton de Vaud; protocol 194/15). Due to the observational design of the study and the fact that the practice of POCUS was already part of the usual care in the ED of the institution, a signed individual informed consent was only required for the use of the data collected for the study. Therefore, in order not to delay the management of the patients, brief verbal information was given to the patient at the beginning of the observation. Full information about the study was then given to the patient as soon as possible. Definite enrollment and data analysis were completed only after individually signed informed consent. If the patient refused to participate, then all study materials were destroyed. No compensation was provided to patients, and all data were anonymized for analysis purposes.

## Results

### In-Training ED Residents

For ED organizational purposes, in-training residents (first or second year of training in internal medicine) were assigned to groups of 6-8 people for a 6-month rotation period. During each 12-hour shift, a resident was responsible for the first-line management of patients with ARF and/or ACF, under the supervision of an emergency medicine specialist. From September 4, 2015, to May 28, 2016 (a total of 268 days; phase 1), 14 residents participated in the observational phase, with no changes to the organization or process of usual care. Twelve interns successfully completed the AURUS training course from May 29, 2016, to September 14, 2016. Thereafter, from September 15, 2016, to February 7, 2018 (a total 511 days; phase 2), they were able to perform an immediate POCUS when managing a patient with ARF and/or ACF, which was the only difference from the observational phase 1.

### Patients

**Figure 3.**
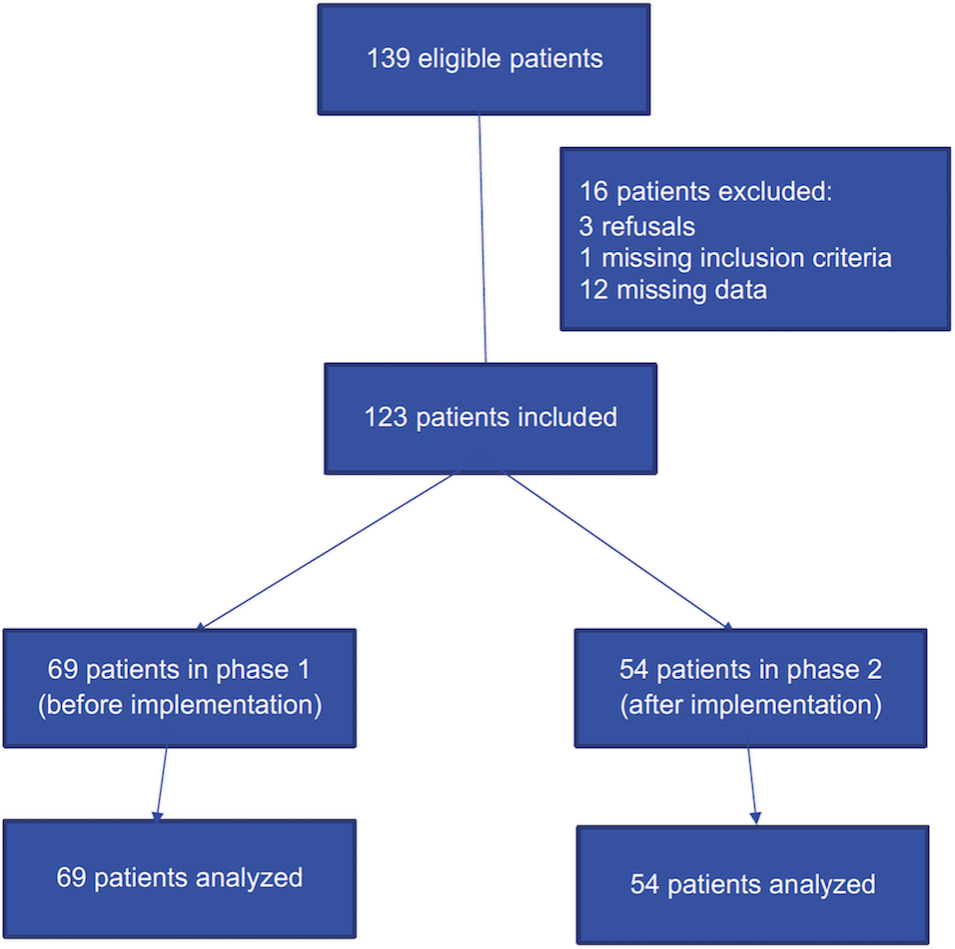
CONSORT (Consolidated Standards of Reporting Trials) study flowchart.

During the whole study period, 139 patients were enrolled, but 3 (2.2%) patients withdrew consent to participate, 1 (0.7%) patient was excluded due to incomplete inclusion criteria, and 12 (8.6%) patients were excluded due to missing data, leaving 123 (88.5%) patients for the analysis (Figure 3). A total of 69 patients were included during phase 1 and 54 patients were included during phase 2. In the final analysis, of the 123 patients, 117 (95.1%) presented with ARF and 20 (16.3%) presented with ACF, of whom 14 (11.4%) presented with a combination of ARF (Figure 3).

The median age of the enrolled patients was 77 (IQR 70‐84) years, and most patients were enrolled for respiratory distress (116/123, 94.3%) and hypoxemia (117/123, 95.1%). The admission characteristics of the enrolled patients are representative of the usual patients with ARF and/or ACF who present to the ED (Table 1).

**Table 1.** Patients characteristics at admission.

	Total population (n=123)	Phase 1 (n=69)	Phase 2 (n=54)
Age (years), median (IQR)	77 (70‐84)	78 (70‐86)	75 (70‐82)
Female sex, n (%)	63 (51.2)	37 (53.6)	26 (48.1)
Prehospital medicalized care, n (%)	19 (15.4)	8 (11.6)	11 (20.4)
Medical history, n (%)			
COPD^a^	35 (28.5)	21 (30.4)	14 (25.9)
Asthma	9 (7.3)	5 (7.2)	4 (7.4)
Ischemic heart disease	41 (33.3)	21 (30.4)	20 (37)
Chronic heart failure	38 (30.9)	17 (24.6)	21 (38.9)
Active or past smoking	44 (35.8)	22 (31.9)	22 (40.7)
Immunosuppressive therapy	4 (3.3)	4 (5.8)	0 (0)
Pulmonary hypertension	7 (5.7)	4 (5.8)	3 (5.6)
Chronic kidney disease	44 (35.8)	22 (31.9)	22 (40.7)
Inclusion criteria, n (%)			
Respiratory distress	116 (94.3)	64 (92.8)	52 (96.3)
Hypoxemia (SpO_2_^b^<92%)	117 (95.1)	66 (95.7)	51 (94.4)
Hypotension (SBP^c^<90 mm Hg)	22 (17.9)	14 (20.3)	8 (14.8)
Clinical hypoperfusion	20 (16.3)	12 (17.4)	8 (14.8)
Admission vital signs, median (IQR)			
SpO_2_ (%)	89 (83‐92)	89 (86‐93)	88.0 (80-92)
Respiratory rate (breaths/min)	28 (24‐32)	28 (25‐32)	28 (24‐34)
Heart rate (beats/min)	100 (87‐117)	100 (88‐115)	105 (85‐126)
SBP (mm Hg)	132 (112‐152)	132 (115‐158)	130 (110‐152)
DBP^d^ (mm Hg)	76 (61‐89)	76 (60‐90)	75 (63‐89)
Laboratory values, median (IQR)			
pH	7.40 (7.35‐7.45)	7.41 (7.35‐7.45)	7.40 (7.36‐7.45)
pO_2_^e^ (kPa)	8.2 (7.1‐9.8)	8.3 (7.4‐10.2	7.7 (6.7‐9.2)
pCO_2_^f^ (kPa)	4.9 (4.1‐6.3)	5.0 (4.4‐6.0)	4.8 (3.9‐6.8)
Lactate (mmol/L)	1.75 (1.40‐2.75)	1.80 (1.40‐2.85)	1.70 (1.40‐2.28)
Creatinine (µmol/L)	104 (73‐151)	108 (73‐152)	98 (74‐148)
Hemoglobin (g/L)	130 (115‐143)	130 (114‐144)	133 (116‐143)
BNP^g^ (ng/L)	398 (185‐924)	267 (164‐680)	566 (311‐1044)
D-dimers (ug/mL)	1392 (643‐2800)	1125 (697‐1437)	2273 (453‐4474)
CRP^h^ (mg/L)	44 (15‐104)	43 (15‐95)	49 (16‐147)

a COPD: chronic obstructive pulmonary disease.

b SpO_2_: oxygen saturation.

c SBP: systolic blood pressure.

d DBP: diastolic blood pressure.

e pO_2_: partial pressure of oxygen.

f pCO_2_: partial pressure of carbon dioxide.

g BNP: brain natriuretic peptide.

h CRP: C-reactive protein.

### General ED Management

The median ED stay duration was 230 (IQR 160‐300) minutes. During their ED stay, of the 123 patients, 98 (79.7%) had a chest x-ray, 40 (32.5%) had a chest CT scan, and 47 (38.2%) had a POCUS performed by a senior supervisor. Pneumonia was the most frequent diagnosis (n=42, 34.1%), followed by acute heart failure (n=41, 33.3%). Antibiotics (n=64, 52%) and diuretics (n=49, 39.8%) were the most frequently prescribed therapies during ED stay. Except for 2 patients (1 death and 1 home discharge), all patients were hospitalized—in half (n=58, 47.2%) of the cases, in the intensive care unit (Table 2).

**Table 2.** Emergency department (ED) management.

	Total population (n=123)	Phase 1 (n=69)	Phase 2 (n=54)
Imaging, n (%)			
Chest x-ray	98 (79.7)	65 (94.2)	33 (61.1)
Thoracic CT^a^	40 (32.5)	21 (30.4)	19 (35.2)
Abdominal CT	14 (11.4)	5 (7.2)	9 (16.7)
Abdominal ultrasound	4 (3.3)	4 (5.8)	0 (0)
Transthoracic echocardiography	3 (2.4)	2 (2.9)	1 (1.9)
POCUS^b^ by senior physician	47 (38.2)	24 (34.8)	23 (42.6)
ED diagnosis, n (%)			
Pneumonia	42 (34.1)	26 (37.7)	16 (29.6)
Acute heart failure	41 (33.3)	19 (27.5)	22 (40.7)
Acute exacerbation of COPD^c^	13 (10.6)	9 (13)	4 (7.4)
Nonpulmonary sepsis	11 (8.9)	8 (11.6)	3 (5.6)
Pulmonary embolism	5 (4.1)	1 (1.4)	4 (7.4)
Pericardial effusion	3 (2.4)	0 (0)	3 (5.6)
Cardiogenic shock	2 (1.6)	1 (1.4)	1 (1.9)
Other diagnosis	6 (4.9)	5 (7.2)	1 (1.9)
Specific ED therapies, n (%)^d^			
Antibiotics	64 (52)	39 (56.5)	25 (46.3)
Diuretic therapy	49 (39.8)	24 (34.8)	25 (46.3)
Bronchodilators	27 (22)	18 (26.1)	9 (16.7)
Noninvasive ventilation	25 (20.3)	15 (21.7)	10 (18.5)
Steroids	17 (13.8)	10 (14.5)	7 (13)
Anticoagulation	14 (11.4)	5 (7.2)	9 (16.7)
Vasopressors	12 (9.8)	6 (8.7)	6 (11.1)
Patient destination after ED stay, n (%)			
Ward	58 (47.2)	36 (52.2)	22 (40.7)
ICU^e^	58 (47.2)	30 (43.5)	28 (51.9)
Other hospital (ICU or ward)	5 (4.1)	2 (2.9)	3 (5.6)
Home	1 (0.8)	1 (1.4)	0 (0)
Death in the ED	1 (0.8)	0 (0)	1 (1.9)

a CT: computed tomography.

b POCUS: point-of-care ultrasound.

c COPD: chronic obstructive pulmonary disease.

d Some patients may have received more than 1 therapy.

e ICU: intensive care unit.

### Comparison Between Phase 1 and Phase 2

The proportion of final diagnoses retained at the end of hospitalization that confirmed the ED diagnosis was 52.2% (36/69) in phase 1 and 94.4% (51/54) in phase 2, a highly significant difference (*χ*^2^_1_=26.146, *P*<.001; Table 3).

**Table 3.** Confirmation of emergency department diagnosis during hospital diagnosis: contingency table^a^.

	Diagnostic confirmed during hospital stay	
	No, n (%)	Yes, n (%)
Phase 1 (n=69)	33 (47.8)	36 (52.2)
Phase 2 (n=54)	3 (5.6)	51 (94.4)
Total (n=123)	36 (29.3)	87 (70.7)

a *χ*2_1_=26.146, *P*<.001.

Compared to phase 1, there was a statistically significant and clinically relevant decrease in the median time to final ED diagnosis in phase 2 (30, IQR 10‐65 min vs 25, IQR 15‐60 min; BF=9.6; Table 4).

**Table 4.** Emergency department (ED) time intervals.

	Phase 1 (n=69)	Phase 2 (n=54)	BF^a^^,^^b^	*P* value^c^
Time to final diagnosis (min), median (IQR)	30 (10‐65)	25 (15‐60)	9.56	.33
Time to final confirmed diagnosis (min), median (IQR)	43 (10‐70)	25 (15‐60)	5.02	.33
Time to administer a correct therapy (min), median (IQR)	70 (20‐120)	47 (25‐101)	1.96	.31
Duration of ED stay (min), median (IQR)	238 (163‐300)	230 (160‐275)	4.18	.42

a BF: Bayes factor.

b Alternative hypothesis: phase 1>phase 2; prior probability: Cauchy, scale 1.0.

c *P* value calculated with the Mann-Whitney *U* test.

When the ED diagnosis was confirmed during the hospital stay, the time to diagnosis in the ED was significantly shorter in phase 2 (25, IQR 15‐60 min vs 43, IQR 10-70 min; BF=5.0), a difference of 18 minutes that is only moderately significant in the Bayesian analysis but clinically highly relevant. Finally, the time to order and start the most appropriate therapy was reduced from 70 (IQR 20‐120) minutes in phase 1 to 47 (IQR 25‐101) minutes in phase 2 (BF=2.0). There was also a reduction in the length of stay in the ED, which was significant in the Bayesian analysis, although probably not clinically relevant (Table 4).

Finally, in-hospital mortality was reduced in phase 2 (3/54, 5.6% vs 9/69, 13% in phase 1), a difference that was highly significant in Bayesian analysis (BF=16.04; Table 5).

**Table 5.** Hospital mortality: contingency table^a^^,^^b^.

	Hospital mortality	
	Alive, n (%)	Dead, n (%)
Phase 1 (n=69)	60 (87)	9 (13)
Phase 2 (n=54)	51 (94.4)	3 (5.6)
Total (n=123)	111 (90.2)	12 (9.8)

a *χ*2_1_=1.93, *P*=.16.

b Bayesian analysis (independent multinomial analysis, with an alternate hypothesis: phase 1>phase 2): Bayes factor=16.04.

Due to the small population sample, we did not perform a formal statistical analysis of patient characteristics, components of ED management, distribution of diagnoses, and therapies administered (Tables1  2). Nevertheless, we demonstrated a substantial decrease in the number of chest radiographs performed during phase 2, with an increase in the number of CT scans performed during the ED stay. In phase 1, according to the study design, a POCUS was performed by a senior attending physician in 34.8% (24/69) of the patients, whereas in phase 2, all patients had a POCUS performed by a junior attending physician, with a second POCUS performed by a senior attending physician in almost half (23/54, 42.6%) of the cases (Table 2).

## Discussion

### Principal Findings

The objective of the IMPULSE study was to investigate the feasibility and impact of implementing a brief, structured training program for ED residents on the management of patients admitted for ARF and/or ACF and their subsequent clinical outcomes. A before-and-after implementation design was selected to emulate the methodology of a randomized controlled trial, while mitigating the potential for contamination bias between the 2 groups. The only difference in the management of patients between the 2 phases was the immediate use of POCUS by the in-training resident in charge in the first-line treatment of the patient. The POCUS training curriculum (AURUS) was chosen for its established presence within the institution and its alignment with the updated recommendations concerning the training objectives of the current guidelines [37,38]. We hypothesized that the immediate use of POCUS by the junior physician after the short AURUS training would improve the diagnostic process, as compared by the later use by a senior physician.

The implementation of the structured, AURUS-based, POCUS program was not only associated with a significantly higher diagnostic accuracy rate but also a shorter delay of diagnosis, particularly when the ED diagnosis was later confirmed during the hospital stay. Our results also suggest that implementing a POCUS training program for in-training residents may be associated with a quicker implementation of the most appropriate therapeutic intervention, and possibly to a reduction in mortality rates, although the study design and the small sample size render the results susceptible to several potential biases. These findings align with those of a previous publication, which demonstrated that the use of POCUS by physicians of varying levels of experience was associated with an improved administration of appropriate therapies, despite no improvement in diagnostic accuracy [40]. This difference in diagnostic accuracy may be due to the more senior level of experience of the involved physicians in the published study, compared to our observation, as the diagnostic contribution of the ultrasound is probably greater for less experienced physicians.

It is also pertinent to consider some of the secondary findings of the IMPULSE study. In both phases of the study, the senior attending physician could conduct a POCUS examination; this occurred in nearly half of the cases in the postimplementation phase, a proportion that exceeds that observed in the preimplementation phase (Table 2). This may have been for verification purposes, but it is also possible that a POCUS conducted by a junior physician may prompt more experienced physicians to perform it with greater frequency, as a ripple effect. Similarly, although this finding should be interpreted with caution, there was a reduction in the number of chest x-rays performed during phase 2 (61.1% of patients only). This suggests that the POCUS may be used in place of this examination. Conversely, the number of CT scans performed during phase 2 was higher, which could be interpreted in two ways. It could be a negative effect of the POCUS, whereby supervisors performed more CT scans to confirm or reject a diagnosis made by their junior colleagues. The observed increase in the number of POCUS examinations performed by supervisors suggests that this may be a more positive effect. POCUS provides a more comprehensive assessment of the clinical situation, leading to a more appropriate use of advanced diagnostic modalities. Subsequent studies will likely address these findings and may confirm these trends, while providing clarification regarding the causes of the observed increase in CT scan use.

Our results show that the reported intervention is not only feasible but also that it has an impact on the clinical management process and possibly on the patient outcome. To the best of our knowledge, these data represent the inaugural demonstration of the clinical impact of a POCUS training program for ED residents. If replicated, they could substantiate the implementation of POCUS in conjunction with history taking and clinical examination by ED residents as a primary diagnostic tool.

### Strengths and Limitations

The IMPULSE study has several notable strengths. The study design reflects the typical circumstances observed in most EDs, wherein patients are initially managed by junior physicians under the guidance of more experienced, senior medical professionals. The characteristics of the included patients and the diagnoses made in the ED demonstrate that this study sample is representative of the population of interest for the use of POCUS, with significant associated morbidity and mortality. The before-and-after study design circumvents the contamination bias observed in several previously published studies. The initial phase reflects the typical practice of most EDs, wherein POCUS is conducted by senior physicians at a relatively late stage, serving as a control for the subsequent postimplementation phase. Interestingly, the rate of inaccurate ED diagnosis during the phase 1 reflects the usual diagnostic accuracy for the management of patients who present to the ED [41-43].

The signal of a clinically relevant impact on the patient outcome is an interesting finding, as morbidity and mortality are the usual end points of choice for ED interventional studies. As POCUS is not a therapeutic procedure, the effect on outcome can only be driven by a quicker and more appropriate administration of efficient therapies. Therefore, our findings of quicker and more accurate diagnosis may explain the reduction of hospital mortality that was evidenced in our small population sample.

It is important to consider the limitations of the IMPULSE study, including the lack of randomization. However, as there is a risk of contamination between the two arms of a randomized controlled trial, we therefore elected to use a before-and-after implementation design as the optimal method to achieve quasi-randomization of patients to limit this risk. A cluster randomization of multiple centers with successive implementation would likely have been the optimal design in this situation; however, it was not feasible to organize. A second limitation is the single-center design and the limited sample of included patients, despite a lengthy recruitment period, particularly in phase 2, with 1 included patient every 9 days. This illustrates the challenges inherent in conducting single-center studies in smaller institutions lacking dedicated clinical research resources. Notwithstanding this significant limitation, the studied population is representative of the typical patients with ARF and/or ACF admitted to the majority of EDs globally, as evidenced by their characteristics and corresponding diagnoses. It would be prudent to reproduce our results in other clinical settings, with the inclusion of a larger sample of patients, before any firm conclusion can be made regarding the impact of implementing a POCUS training program for in-training ED residents. These limitations do not affect the fundamental conclusions of the presented results.

### Conclusion

In conclusion, the IMPULSE study demonstrates that a brief, structured training program for ED residents is both feasible and enables them to use POCUS as a primary tool for the initial management of patients presenting with ARF and/or ACF. The deployment of POCUS by these less experienced physicians may be associated with an increase in diagnostic accuracy, comparable to that observed in published data on POCUS use by experienced ED physicians. Furthermore, it may be associated with a reduction in the time required for in-training residents to reach a correct diagnosis and with a more rapid and appropriate prescription of a specific therapy, which may result in a decrease in hospital mortality. The results of the IMPULSE study also validate the AURUS training curriculum, demonstrating that this structured, stepwise approach to training is not only feasible but also efficient. These results must be replicated and validated in other settings with larger patient samples. However, the methodology presented herein is appropriate for limiting the issues of blinding and randomization in the study of such diagnostic tools and may be used by future studies.

## Abbreviations:

ACF: acute circulatory failure
ARF: acute respiratory failure
AURUS: Association des urgentistes et réanimateurs intéressés à l’ultrasonographie
BF: Bayes factor
CT: computed tomography
ED: emergency department
IMPULSE: Impact of a Point-of-Care Ultrasound Examination
POCUS: point-of-care ultrasound
